# Mr. Dunn, on the Internal Use of the Arsenic Acid

**Published:** 1808-12

**Authors:** John Dunn

**Affiliations:** Wells, Norfolk


GENTLEMEN,

IT is a melancholy but well-established fact, (says Mr. Roberton, when treating on Cantharides) that the violence of opposition, which any improvement either in surgery or medicine meets with, is too often exactly in proportion to its importance in the removal of a disease. Instances of this kind may be fully exemplified, relative to the internal exhibition of arsenic. That it undermines the constitution, and lays a foundation for mortal diseases is an objection so forcible and universal, that even practitioners who are fully acquainted with its efficacy, cannot but administer it with many anxious apprehensions. I have observed, and heard it frequently remarked, that swellings of a dropsical nature have oftentimes succeeded the use of this oxide. But whether these appearances, whether the glassy eye, the bloated countenance, or the general debility, are in consequence of the arsenic, is, I must confess, a matter of extreme ambiguity. Intermittents, however treated, generally leave some ravages. Indeed it is almost an invariable custom to give tonics after the secession of any kind of fever; and for what? but to remove the excessive weakness, to contract the relaxed and enervated fibre, and give tone and energy to the nearly exhausted circulation. As the stimulating nature of arsenic is irrefragable, and as all stimulants promote absorption, that mercury, and even digitalis, as lately demonstrated by Mr. Saunders, owe their absorbent entirely to their exciting properties, so far then, if we may judge by analogy, from arsenic laying the foundation of a dropsy, it must inevitably tend to remove it. But time and experience undoubtedly will decide. The oppositions advanced, are made by gentlemen of observation and candour, and it is to their further examination of this interesting problem, we must alone look to for certainty and conviction.

Philosophers have asserted, and on the testimony of experiment, that the action or activity of metals depends on the proportion of oxygen with which they are united, and on on this principle we may attribute the more immediate influence of the muriat of mercury to the submuriat.

Having about six weeks back, two cases of obstinate intermittents, which resisted the usual means employed, as the bark, canella albe, Fowler's mineral solution, emetics, &c. I resolved upon the trial of the arsenic acid. The acid was made, according to Bucholz's process, by first mixing in a retort, one part of muriatic acid, four parts of the white oxide of arsenic or arsenicus, and twelve parts of nitric acid; then boiling the mixture till nitrous gas ceases to be engaged. After the mass was evaporated to dryness, by exposing it for a few minutes to a lowered heat, I dissolved it, being about 32 grs. in 8 oz. of boiling water. Of this solution, I gave three drops every two hours, combined with either an equal quantity of tincture of opium, or cascarilla bark. The result was particularly successful. The following are facts which at once prove the fallacy of its being injurious, (at least when given in small doses) and at the same time its decided and remarkable efficacy.

Robert Curle, a boy about 14 or 15 years of age, who had lately enjoyed exceeding good health, was attacked in the commencement of September with general rigour, great prostration of strength, and a vomiting of green bilious matter; these symptoms were gradually superseded by the hot stage of fever; the pyrexia was considerable, tongue parched, and skin excessively hot and dry; he took during this stage, a draught with nitre, which was immediately rejected. He then attempted to swallow a beverage he called for, made with apple-juice and water, but the irritability of his stomach continued so great as to refuse every thing taken. During the remission of the paroxysm, he took an emetic, which operated perfectly well; and after his stomach was easier, he began with three drops of Fowler's solution, which were repeated every two hours. The second day following, he was attacked as usual, and the hot fit, if any thing, was more violent. His medicine was continued with the addition of one, and afterwards two drops at a dose. Notwithstanding, the paroxysms came on every other day equally severe for nearly a fortnight, when they remitted for about three days. A relapse ensued, the type, however, was more regular. He then began with three drops of the arsenic acid, which was afterwards increased to four. This dose was taken every two hours. The next day he was again attacked, but with much less severity. Since this time, the fever returned no more, and he is now as hearty and free from disease as ever.

Encouraged

Encouraged by this success, I next gave it a trial in a case of Rheumatism. The subject's name was Ely, of the parish of Wighton. On her first application, she complained of considerable pain and rigidity of her arm. The tendons at the elbow-joint were very much enlarged, and the muscles of her arm were so contracted, that she could scarcely move it. A strong volatile liniment was ordered, frequent friction with flannel, and the topical application of the warm bath.

On the 7th of Sept. which was about three days after, she was much worse; her arm the same; much pain and inflammation about the ancles; pulse quick; tongue white, and indeed she had now every symptom of acute rheumatism. Leeches were applied to her ancles, the inflamed parts were ordered to be kept constantly wet with a lotion composed of spt. terebinth. et aqua. The sudorific regimen was enjoined.

℞. P. ipecac. comp. gr. vj. Pulv. quaq. quart. hor sumend. P. ipecac. c. gr. viij. Calomel gr. $\frac{1}{4}$. G. a. q. s. ft. bol. h. s. sumend.

As her bowels had been rather relaxed, no aperient was deemed requisite. In a few days the inflammatory symptoms abated, but the pain and tension still continued, and every joint was alternately affected.

℞. Solut. Fowler miner. Tinct. opii aa. ʒij. sumat. gtt. viij. secund. hor.

Sept. 10. Continues much the same as to pain and rigidity of the joints. Rep. gutta also.

℞. P. cort. fl. ʒß. P. canell. alb. gr. v. M. Pulv. sumat. ter in die.

17th. Little amendment, very weak, still much irritability and contraction of the muscles.

℞. Solut. arsenic acid gtt. iij. quoq. bihor.

19th. Better. Rep. gutta.

21st. The arm is reduced to its natural size; her ancles, though painful, are considerably better.

24th. Feels so much recovered, that she complains now only of weakness, which, perhaps, was aggravated by her taking, from some serious mistake, a quantity of white vitriol to the amount of a scruple.

℞. Vin. ferri ʒvj. sumat. cochl. parv. j. ter in die.

The last words which I heard from her, was, that she is very finely.

On the 16th of Sept. George Parker, residing in Wells, complained, " his poor boy, who had got the illness that is about, about, wanted some medicine, as he had been sadly with it off and on," as he expressed himself, "for some time."

℞. Antim. tart. gr. j. P. ipecac. gr. v. M. pulv. Stat. sum. Solut. arsenic acid. Tinct. opii aa. ʒj. M. sumat. gtt. v. quaq. secund. hor.

The next day he was again attacked, but with a very transient paroxysm. Though transient, it was his last, and he is now quite well.

A few days after, Geo. P. himself came, and with the same complaint as his son's was. The type of his intermittent was a regular quotidian. His head suffered excruciating pains, and when I saw him, his skin was almost scorched with heat; pulse quick; frequent nausea; dry tongue; and bowels very irregular. I gave him an emetic with a scruple of ipecacuanha, and afterwards ordered eight drops of the compound solution, of equal parts of arsenic acid and tinct. of opium, to be taken every two hours. The day he applied was the 26th of September.

On the 29th, I found he had entirely lost his fever. Being rather of a phthisical habit, and at that time labouring under a troublesome cough, the following pills were prescribed.

℞. Fer. vit. ℈ij. Pil. scillæ ʒij. P. opii gr. iij. M. et divid. in pilula xl. Capiet iij. ter in dies.

His health is now perfectly re-established.

On the 18th of October, a man by the name of Lake, of the parish of Holkham, who had been labouring under a tertian intermittent for a considerable time, applied for medical assistance. He had been previously attended by a Mr. R. and taken about thirty-two bark powders, but without deriving the least benefit. On the first day of his application, he began with the arsenic solution without any emetic, *ut* infra præscripta.

℞. Solut. arsenic acid. Tinct. cascarillæ æ ʒiij. M. sumat gtt. vj. quaq. dua hor.

On the 22d, the drops were repeated, but he informed me the fever had attacked him only once since he took the medicine. I saw him again on the 26th, and with ineffable joy in his countenance, he told me that he has suffered nothing from his complaint since the time above mentioned. My appetite says he, is improved, and my strength so much recruited, that I feel quite another man. To these might be added, no less than twenty other instances where I have given it without the least injury, but with the most decided advantage. One patient, however, took it to the extent of nine drops, which brought on a great degree of vertigo; but another, (like the first) imagining the larger the dose the sooner sooner the cure, took at least sixteen drops at once, and without any bad consequence. To avoid these errors in future, the drops were given in a mixture; and though the latter, a Mrs. Norman, had taken that extraordinary dose without any sensible effect, I found in three days after, the diminished dose of four drops every two hours effected a cure.—Such are the results of an acid, which, I believe, has never till now been ventured internally; if I am wrong in this conclusion, I hope to be forgiven. To calculate on the superior advantages of this solution is useless; what has been said of the white oxide, may be said of this acid, only that the latter is a more active preparation. The value of a medicine is generally in proportion to the simplicity of its form. Though the bark may cure when taken in sufficient quantity, any intermittent; yet, the disgust which it creates, the nausea which it excites, and the neglect of it which consequently ensues; two-thirds of the sick, rather than take it, will leave themselves a prey to their disorder, trusting to the chances of Fortune, or the auspices of their Creator.

When we consider the importance of man, either in public or domestic life, when we see a parent surrounded by a herd of children, craving for bread, and he himself incapable of providing it, the practitioner, (or in truer words the empiric,) who leaves him, who deserts him, because the poor invalid cannot swallow his favourite drug, ought to be branded with infamy, and hated as an unfeeling barbarian. As such, Gentlemen, I consider every improvement, either in the formula of medicines, or in the substitution of agreeable and more concentrated remedies for the complicated and more disgusting, of essential advantage to mankind, I therefore submit the above, with every degree of diffidence and respect, from Yours, &c.

JOHN DUNN.

*Wells, Norfolk, Oct. 27,* 1808.

P. S. It is needless, perhaps, to add, that particular attention should be paid in the degree of heat made use of, during the evaporation of the acid to dryness.

*Observations*

